# Nutzung digitaler Informations- und Unterstützungsangebote durch pflegende An- und Zugehörige aus Sicht von pflegefachlichen Gutachter:innen: Ergebnisse einer Fokusgruppenstudie

**DOI:** 10.1007/s00391-025-02503-8

**Published:** 2025-10-02

**Authors:** Annika Hudelmayer, Marie-Christin Redlich, Johanna Schütz, Florian Fischer

**Affiliations:** Bayerisches Zentrum Pflege Digital, Hochschule Kempten, Albert-Einstein-Str. 6, 87437 Kempten, Allgäu Deutschland

**Keywords:** Informelle Pflege, Pflegebedarf, Pflegende Angehörige, Digitale Angebote, Häusliche Pflege, Informal care, Care needs, Family caregivers, Digital information, Home care

## Abstract

**Hintergrund:**

Zu Beginn einer pflegerischen Versorgung besteht bei Menschen mit Pflegebedarf (MmP) und deren An- und Zugehörigen ein besonders hoher Informationsbedarf. Ziel dieser qualitativen Studie war es, eine Einschätzung zur Nutzung digitaler Informations- und Unterstützungsangebote durch informell Pflegende aus Perspektive von Gutachtenden des Medizinischen Dienstes Bayern (MD Bayern) zu erhalten, welche Hausbesuche in großer Zahl durchführen.

**Methode:**

Von Juni bis Juli 2024 wurden vier leitfadengestützte, onlinebasierte Fokusgruppeninterviews mit 29 pflegefachlichen Gutachtenden des MD Bayern durchgeführt. Das Datenmaterial wurde transkribiert, pseudonymisiert und mittels qualitativer Inhaltsanalyse ausgewertet.

**Ergebnisse:**

Die Erfahrungen der Gutachtenden verdeutlichen, dass digitale pflegebezogene Angebote von MmP trotz bestehenden Bedarfs nur wenig genutzt werden. Unter An- und Zugehörigen ist die Nutzung eher verbreitet, insbesondere sogenannte Pflegegradrechner. Als Gründe für die geringe Nutzung vermuten die Fachpersonen das höhere Alter, mangelnde digitale Kompetenzen sowie eine eingeschränkte Infrastruktur in ländlichen Gebieten. Die Gutachtenden bewerten einen Informationsflyer als geeignetes Mittel, um pflegende Haushalte gezielt über digitale Angebote aufzuklären.

**Diskussion:**

Um die Nutzung digitaler Angebote im Kontext der häuslichen Pflege zu erhöhen, sind gezielte Informationskampagnen sowie Förderungsmaßnahmen digitaler Kompetenzen unerlässlich. Auch eine stärkere Ausrichtung dieser Angebote an den heterogenen Bedarfen der Zielgruppe ist erforderlich.

## Abstract (en)

**Background:**

People in need of care and their relatives have a high need for information, especially at the beginning of a need for care. The aim of this qualitative study was to explore the use of digital information and support services by informal caregivers from the perspective of medical assessors at the Bavarian Medical Service (MD Bayern), who conduct numerous home visits.

**Method:**

From June to July 2024, four guided online-based focus group interviews were conducted with 29 medical assessors from MD Bayern. The data were transcribed, pseudonymized, and evaluated using qualitative content analysis.

**Results:**

The medical assessors’ experience show that digital care-related services are only used to a limited extent by people in need of care, despite the existing need. Use is more widespread among relatives, especially the so-called “care degree calculator”. The experts believe that the main reasons for the low use are older age, lack of digital skills, and limited infrastructure in rural areas. Experts assessed an information flyer as a suitable means of informing caregiving households specifically about digital services.

**Conclusion:**

In order to increase the use of digital services in the context of home care, targeted information campaigns and measures to promote digital literacy are essential. In addition, these services need to be more closely aligned with the heterogeneous needs of the target group.

## Hintergrund und Studienziel

Ende des Jahres 2023 belief sich die Anzahl der Menschen mit Pflegebedarf (MmP) in Deutschland auf etwa 5,7 Mio. Mit etwa 86 % wird der größte Anteil der MmP im häuslichen Bereich versorgt [23]. Verschiedene Studien zeigen, dass MmP und ihre An- und Zugehörigen einen hohen Informations- und Unterstützungsbedarf aufweisen, insbesondere in der Anfangsphase der pflegerischen Versorgung [3, 10, 18]. In dieser Phase ist der Bedarf an leicht zugänglichen und grundlegenden Informationen besonders ausgeprägt [10]. Dennoch werden bestehende Informations- und Unterstützungsangebote aufgrund unzureichender Bekanntheit [8], Unübersichtlichkeit der Angebote oder Zeitmangel [3, 10] nur wenig genutzt. Weitere Hindernisse bestehen in der dynamischen Marktentwicklung sowie in der teils unklaren Qualität und Herkunft der Informationen [4, 13, 20].

Die Pflegebegutachtung stellt einen Weg dar, um die Zielgruppe frühzeitig zu erreichen und über entsprechende digitale Angebote zu informieren. Da Pflegebegutachtende eine Multiplikator:innenfunktion einnehmen könnten, wird im Rahmen dieses Beitrags der Frage nachgegangen, wie die Pflegebegutachtenden die Nutzung von Online-Angeboten (z. B. Webseiten und Apps) sowie darauf einflussnehmende Faktoren in der häuslichen Pflege mit Blick auf das Alter(n) einschätzen.

## Studiendesign und Methoden

Es wurden leitfadengestützte Fokusgruppeninterviews mit Gutachtenden des Medizinischen Dienstes (MD) Bayern im Juni und Juli 2024 onlinebasiert via Cisco Webex (Cisco Systems GmbH, Garching) durchgeführt. Insgesamt fanden vier Fokusgruppen mit einer Dauer von jeweils 60 bis 90 min sowie je 6–8 Teilnehmenden statt. Die Rekrutierung erfolgte durch den MD Bayern. Zwei Interviewerinnen und eine Protokollantin waren bei allen Interviews anwesend.

Es wurde ein teilstrukturierter Interviewleitfaden mit fünf Themenbereichen in Anlehnung an das themenzentrierte Interaktionsmodell von Cohn von einem interdisziplinären Studienteam (Sozial- und Gesundheitswissenschaftler:innen ohne Vorkontakte mit den Teilnehmenden) entwickelt [2].

Die aufgezeichneten Interviews wurden mit der Software f4transkript (dr. dresing & pehl GmbH, Marburg), in Anlehnung an die inhaltlich-semantische Transkription verschriftlicht und von einer Projektmitarbeiterin überarbeitet [7]. Nach einer Pseudonymisierung erfolgte die Auswertung der Daten basierend auf der inhaltlich strukturierenden qualitativen Inhaltsanalyse nach Kuckartz, wobei die qualitative Datenanalysesoftware MAXQDA 2022 (VERBI GmbH, Berlin) verwendet wurde [14]. Die Interpretation der Ergebnisse erfolgte von zwei Autorinnen, um eine konsensuelle Validierung der Kategorien sicherzustellen. Die Ergebnisdarstellung orientiert sich an den Standards for Reporting Qualitative Research [16].

## Ergebnisse

Zur Analyse werden vier Fokusgruppen mit insgesamt 29 Gutachtenden des MD Bayern herangezogen (17 Frauen, 12 Männer, Altersspanne: ca. 30 bis 60 Jahre). 24 Gutachtende verfügen über eine Qualifikation in der Gesundheits- und Krankenpflege oder Altenpflege. Die teilnehmenden Gutachtenden sind durchschnittlich seit 6,6 Jahren (Spannweite: 10 Monate bis 24 Jahre) beim MD Bayern tätig.

Die Ergebnisse dieses Beitrags konzentrieren sich auf die Einschätzung der Gutachtenden zu Nutzungsunterschieden digitaler Angebote und Aspekten der (Nicht‑)Nutzung dieser Angebote in der häuslichen Pflege^1^.

### Nutzungsunterschiede digitaler Angebote in der häuslichen Pflege

Die Gutachtenden schildern aus ihrer Sicht, dass pflegebezogene digitale Angebote in Haushalten von MmP aufgrund des Alters selten genutzt werden. Auf Nachfrage wird auf Unterschiede in der Nutzung zwischen den verschiedenen Haushaltsakteur:innen verwiesen.

#### Nutzung digitaler Angebote durch MmP

Das geringe Level der Digitalisierung der Pflegesituation führen die Gutachtenden auf das fortgeschrittene Alter der MmP zurück, das sie mit eingeschränkten digitalen Vorerfahrungen, Fähigkeiten und Interessen dieser spezifischen ‚Generation‘ in Verbindung bringen. Auch körperliche und kognitive Einschränkungen werden als Gründe für die geringe Nutzung digitaler Angebote genannt. Ein höheres Alter gehe oft mit einem schlechteren Gesundheitszustand einher, der die Nutzung digitaler Technologien erschweren könne. In einigen Fällen könnten die Einschränkungen, aufgrund welcher der Pflegegrad beantragt wird, die Anwendung digitaler Hilfsmittel verhindern. Gleichzeitig bieten technische Assistenzsysteme in solchen Kontexten potenzielle Unterstützung, was die Nutzung fördern könnte.

Gegensätzlich wird jüngeren MmP eine stärkere Nutzung zugeschrieben – insbesondere von Assistenzsystemen für spezifische Einschränkungen. Erfahrungen im Beruf mit Online-Aktivitäten und ein höherer Bildungsstand werden als förderliche Aspekte hinsichtlich der Nutzung digitaler Hilfsmittel genannt.

Informationen würden von MmP primär über persönliche Kontakte wie Bekannte, die sich in einer vergleichbaren Lebenssituation befinden, sowie Nachbarschaft und kirchliche Gemeinschaften gewonnen. Trotz der geringen Nutzung digitaler Informationsangebote zeige sich, dass Smartphones und Tablets zunehmend zur Aufrechterhaltung sozialer Kontakte eingesetzt werden, insbesondere zur Familie:

„Auch die Senioren, wo ich vorhin gesagt habe, die, soziale Medien, Tablet, Smartphone oder so nutzen, um eben Kontakte aufrechtzuerhalten, […] weiß gar nicht, ob es überhaupt schon mal vorgekommen ist, dass sich da jemand bezüglich der Begutachtung informiert hat. Eher, dass sie wenig informiert sind“ (FG2, Pos. 82).

In dem Zitat wird neben der Nutzung digitaler Medien zur Aufrechterhaltung von Kontakten ein weiterer Aspekt benannt, dem von den Gutachtenden in den Fokusgruppen ein hoher Stellenwert zugeschrieben wird. Bei einem Großteil der Antragstellenden nehmen die Gutachtenden im Rahmen des Hausbesuchs große Informationsdefizite wahr. Diese können aus deren Sicht nicht (ausschließlich) durch digitale Angebote gedeckt werden. Um diese Bedarfe zu adressieren, scheinen, wie an anderer Stelle ausgeführt wird, vielfältige Informationsstrategien erforderlich.

Neben der Möglichkeit, mithilfe digitaler Geräte Kontakte aufrechtzuerhalten, nutzt die Gruppe der MmP diese außerdem als Notrufsystem oder vereinzelt zur Unterstützung beim Einkaufen.

#### Nutzung digitaler Angebote durch pflegende An- und Zugehörige

In den Fokusgruppen legten die Gutachtenden erwartungsgemäß den Fokus auf die MmP. Da pflegende Angehörige aber an einem Großteil der Begutachtungen teilnehmen, teilten sie auf Nachfrage auch ihre Wahrnehmung zur Nutzung digitaler Angebote durch Angehörige. Auch hier spielt das Alter eine wichtige Rolle: Jüngere Angehörige würden häufiger digitale Angebote nutzen als etwa Partner:innen der MmP:

„Das sind dann vielleicht eher dann ihre Kinder oder Enkel, die da jetzt so langsam natürlich auch besser wie wir damit reinwachsen und aufwachsen“ (FG4, Pos. 41).

Mit Blick darauf, wofür digitale Angebote im Pflegekontext von den Angehörigen vornehmlich genutzt werden, zeichnen sich Pflegegradrechner^2^ als ein Tool ab, das zur Vorbereitung auf den Begutachtungsprozess genutzt und das jeweilige Ergebnis ausgedruckt mitgebracht wird:

„Was mir immer begegnet, sind irgendwelche selbst ausgedruckten Pflegegradrechner, wo die Angehörigen auf die entsprechenden Homepages zugreifen“ (FG3, Pos. 81).

Auch die Online-Recherche zu Pflegediensten und digitale Absprachen über Messenger-Dienste wie WhatsApp seien üblich. Spezialisierte Pflege-Apps begegnen den Gutachtenden hingegen selten.

### Aspekte der (Nicht‑)Nutzung digitaler Informations- und Unterstützungsangebote

Die Gutachtenden heben hervor, dass förderliche und hinderliche Aspekte für die Nutzung digitaler Angebote im Rahmen der Begutachtungen kaum explizit thematisiert werden, sodass die Schilderungen überwiegend auf ihren Beobachtungen beruhen.

#### Personenbezogene Aspekte

Wie bereits dargelegt, betonen die Gutachtenden im Zusammenhang mit dem MmP und dessen An- und Zugehörigen das Alter als wichtigen Aspekt für die Nicht-Nutzung digitaler Angebote. Als förderliche Aspekte für die Nutzung von digitalen Angeboten werden bei allen Beteiligten das jüngere Alter sowie der Umgang mit Digitalisierung im Berufsleben hervorgehoben. Darüber hinaus wird auch der Bildungsstand als Einflussfaktor genannt:

„Wenn Angehörige einen höheren Bildungsgrad haben, dann lesen sie sich auch mehr ein, sind motivierter und nutzen auch mehr digital“ (FG4, Pos. 321).

Ein zentraler Aspekt, der sich in den Fokusgruppen abzeichnet, ist das Verhältnis bzw. die Beziehung zwischen den pflegenden Angehörigen und den Personen mit Pflegebedarf sowie die damit einhergehenden Erwartungen an den Umfang und die Dauer einer Pflegesituation.

So legen die Gutachtenden dar, dass die Angehörigen der MmP eine zentrale Rolle in Bezug auf die Nutzung digitaler Angebote einnehmen. Sie schaffen häufig für die MmP die notwendigen technischen Voraussetzungen zur Nutzung digitaler Angebote, etwa durch die Installation von Routern und die Zurverfügungstellung von mobilen Endgeräten. Zudem sind sie bei Fragen oder Problemen erste Ansprechpersonen. Dabei verfolgen die Angehörigen zwei Ziele: Einerseits möchten sie selbst mit den Personen mit Unterstützungsbedarf in Kontakt treten und ihnen soziale Interaktion und Teilhabe ermöglichen. Andererseits möchten sie die Erreichbarkeit von Notfallkontakten sicherstellen, um deren Sicherheit zu gewährleisten und sich selbst zu beruhigen.

Die angedeutete Erwartung an die Pflegesituation zeichnet sich insbesondere mit Blick auf Eltern von Kindern mit Pflegebedarf ab. Im Vergleich zu pflegenden Angehörigen älterer Personen nutzen sie digitale Angebote häufiger und intensiver, was nur zu Teilen auf deren jüngeres Alter zurückgeführt wird. So legen die Gutachtenden dar, dass die Eltern meist sehr gut auf die Begutachtung vorbereitet sind und über eine hohe Expertise in Bezug auf die Diagnose und Behandlung ihrer Kinder verfügen. Zudem seien sie häufig in Online-Foren vernetzt und nutzen Selbsthilfegruppen, insbesondere bei seltenen Erkrankungen, um sich überregional mit anderen Betroffenen auszutauschen. Die Gutachtenden erklären dies unter anderem damit, dass die Eltern in diesen Kontexten fast immer intensiv an dem Versorgungsprozess beteiligt sind.

Als weiterer Aspekt wird von den Gutachtenden die Sorge vor Online-Missbrauch und Betrug als Hindernis für die Nutzung digitaler Angebote wahrgenommen. Dies betrifft insbesondere Personen mit wenig Erfahrung in der Internetnutzung. Diese Sorge bezieht sich nicht speziell auf Online-Tätigkeiten im Kontext der Pflege, sondern allgemein auf Aktivitäten im Internet.

#### Personenunabhängige Aspekte

In allen Fokusgruppen werden auch personenunabhängige Aspekte genannt, die beispielsweise strukturell verankert sind, z. B. fehlende technische Infrastruktur, insbesondere in ländlichen Gebieten:

„Ich war zwar letzte Woche in einem kleinen Dörfchen […], kein Empfang. […]. Wenn dann ein paar Bilder geladen werden müssen, wo sie dann schon eine halbe Stunde warten müssen“ (FG2, Pos. 114).

Zudem wird die fehlende Passgenauigkeit der Angebote zu den spezifischen Bedürfnissen der Zielgruppe (z. B. persönliche Ansprache und soziale Interaktion) als Grund für die Nicht-Nutzung identifiziert. Dies zeigt sich beispielsweise am Wunsch der Zielgruppe, online verlässliche Informationen zur Verfügbarkeit regionaler Angebote zu erhalten. Bisher wird dieser Wunsch in digitalen Angeboten allerdings kaum umgesetzt.

Abschließend legen die Gutachtenden übereinstimmend dar, dass sie einen Informationsflyer für ein geeignetes Medium halten, mit dem Pflegende und ihre Angehörigen frühzeitig informiert werden können.

## Diskussion

Die Ergebnisse dieser Studie bieten wertvolle Einblicke aus der Perspektive der MD-Gutachtenden in Bezug auf die Nutzung digitaler Pflegeangebote im häuslichen Pflegekontext. Trotz des hohen Informationsbedarfs der Zielgruppe zeigt sich, dass die Nutzung digitaler Angebote bislang gering ist. Stattdessen scheinen MmP sowie ihre An- und Zugehörigen analoge Informationsquellen zu bevorzugen, was auch auf eine mangelnde Bekanntheit digitaler Möglichkeiten hindeuten könnte. Die Gutachtenden identifizieren aus ihrer Sicht das fortgeschrittene Alter bzw. fehlende Erfahrungen mit digitalen Technologien als primären Faktor für die geringe Nutzung. Häufig wird dabei verallgemeinernd angenommen, dass mit zunehmendem Alter ein geringeres technisches Interesse, weniger digitale Kompetenzen sowie eine niedrigere Vertrautheit mit digitalen Angeboten einhergehen. Die Beobachtungen der „digitalen Ungleichheit“ decken sich mit früheren Studien, die ähnliche Barrieren hervorheben [24, 25]. Daran anschließend stellt sich die Frage, wie MmP und ihre Angehörigen auf digitale Informationsangebote hingewiesen werden können. Obwohl der analoge Flyer von den Gutachtenden als geeignetes Medium wahrgenommen wird, legen sie in den Fokusgruppen dar, dass viele MmP mit der hohen Informationsdichte im Vorfeld der Begutachtung überfordert sind oder bereits zu einem früheren Zeitpunkt grundlegende Informationen benötigt hätten. Aufgrund der Heterogenität der Zielgruppe sind vielfältige Informationsstrategien notwendig. Die Komplexität und Vielfalt der Wege in eine Pflegesituation bzw. bis zur Beantragung eines Pflegegrades, erschweren diesen Prozess [9].

Die Aussagen in den Fokusgruppen zeigen eine homogene Wahrnehmung von MmP und ihren Angehörigen, die die Heterogenität innerhalb dieser Gruppe überdecken. Erst durch gezieltes Nachfragen wird deutlich, dass jüngere Pflegende, insbesondere jene, die sich um Kinder mit Pflegebedarf kümmern, einen höheren Grad an digitaler Informiertheit und Vernetzung aufweisen. Dies lässt sich auf unterschiedliche Aspekte zurückführen. Während bei einem Unterstützungsbedarf im Alter oft unklar ist, wie lange dieser anhält, wird bei der Versorgung eines Kindes mit Pflegebedarf von einer langfristigen Dauer ausgegangen [11]. Gleichzeitig haben die Kinder oft spezielle und komplexe Diagnosen, die eine spezifische Behandlung erfordern, sodass die Zahl an Personen mit vergleichbaren Einschränkungen gering ist [12]. Um Kontakte zu Menschen in ähnlichen Lebenssituationen herzustellen, bedarf es daher einer gezielten Vernetzung, die zu anderen Familien häufig über Foren oder anderen digitalen Räumen stattfindet. Auch den Gutachtenden zufolge sind die Eltern von Kindern mit Pflegebedarf online sehr gut vernetzt.

Ein weiterer Unterschied zwischen diesen beiden Pflegekonstellationen könnte die jeweilige Beziehung zwischen Angehörigen und MmP sein. Die Beziehung zu den Eltern mit Pflegebedarf zeichnet sich insbesondere dahingehend aus, dass (zumeist) viele Jahre vorausgehen, in welchen andere innerfamiliäre Unterstützungs- und Abhängigkeitsverhältnisse prägend waren [1]. Diese besteht bei Kindern mit Pflegebedarf nicht. Aufgrund der unterschiedlichen Schwerpunktsetzung der Fokusgruppen wird dies nur an einzelnen Stellen deutlich. Hier scheinen weiterführende Untersuchungen gewinnbringend, um Differenzierungsmerkmale und mögliche Potenziale abzuleiten.

Als entscheidender Aspekt in Bezug auf die Nutzung digitaler Angebote der MmP kristallisiert sich das jeweilige Engagement der pflegenden Angehörigen heraus. Sie agieren häufig als ‚Enabler‘. Neben der Bereitstellung der Infrastruktur zeigt sich in anderen Studien, dass sie auch erste und oft wichtige Ansprechpersonen sind, wenn es um das Erlernen der entsprechenden Kompetenzen geht [26]. Die Pflegekonstellation und familiäre Unterstützung haben somit maßgeblichen Einfluss auf die Verbreitung und Nutzung digitaler Angebote in der häuslichen Pflege [17].

Eine in den Ergebnissen hervorgehobene Barriere ist die teilweise unzureichende digitale und materielle Infrastruktur in ländlichen Räumen, die den Zugang zu digitalen Angeboten einschränken kann. Auch wenn diese Problematik beschrieben wird, ist zu berücksichtigen, dass der Ausbau der Breitbandinfrastruktur in Deutschland in den letzten Jahren auch im ländlichen Raum deutlich vorangeschritten ist [6]. Dabei bleibt die Breitbandversorgung in ländlichen Gebieten Bayerns häufig hinter der in städtischen Regionen zurück [15]. Statt von einer flächendeckenden Barriere auszugehen, sollten die Aussagen dementsprechend differenziert eingeordnet werden. Dennoch können die lokalen Unterschiede langfristig den Nutzen und die Verbreitung digitaler Angebote beeinträchtigen und Aspekte der digitalen Ungleichheit verstärken [5, 19].

### Limitationen

Zunächst ist darauf hinzuweisen, dass das Thema Digitalisierung nicht Gegenstand einer Pflegebegutachtung ist. So wurden die Gutachtenden zu einem sehr spezifischen Thema befragt, das in ihrer täglichen Arbeit bei der Begutachtung nur eine untergeordnete Rolle spielt. Die Begutachtung ist als soziale Interaktion zu verstehen, die nicht losgelöst von gesellschaftlichen Annahmen stattfindet und in den Aspekten soziale Erwünschtheit im Auskunftsverhalten sowie Unterschiede in der Selbst- und Fremdwahrnehmung eine wesentliche Rolle spielen [21, 22].

Zudem liegt der Fokus der Gutachtenden auf der Person mit Pflegebedarf, während die Angehörigen nur am Rande berücksichtigt werden und das weitere soziale Umfeld unberücksichtigt bleibt. Wahrnehmungen und Einschätzungen zur Nutzung digitaler Angebote durch Angehörige erfolgten daher allenfalls auf Nachfrage und in begrenztem Umfang.

Hinsichtlich der Auswahl und Rekrutierung der Teilnehmenden ist anzumerken, dass diese nicht im Einflussbereich des Studienteams lagen, sondern durch die Verantwortlichen des MD Bayern festgelegt wurde.

## Fazit für die Praxis

- Zielgruppenspezifische digitale Pflegeangebote sind MmP und ihren Angehörigen weitgehend unbekannt. Eine universelle Lösung zur Vermittlung von Informationen gibt es für die heterogene Zielgruppe nicht – daher sind vielfältige Informationsstrategien erforderlich.
- Angehörige nehmen eine zentrale Rolle als ‚Enabler‘ bei der Nutzung von digitalen Angeboten ein und sollten gezielt über bestehende digitale Angebote informiert werden.
- Der Versand eines klassischen Informationsflyers im Vorfeld einer Pflegebegutachtung ist ein geeigneter Zugangsweg, um MmP und Angehörige frühzeitig zu erreichen und die digitalen Angebote bekannter zu machen. Dennoch bestehen weiterhin Nutzungsbarrieren, die es gezielt zu adressieren gilt.

## Data Availability

Da die Daten nicht vollständig anonymisiert werden können, ist eine Einsicht der empirischen Originaldaten nicht möglich.

## Footnotes

1 Weitere Themenbereiche des Leitfadens waren: Wahrnehmung des Informationsflyers, Potenziale und Grenzen der Angebote, Zukunftsperspektiven sowie Möglichkeiten zu digitalen Technologien in der Begutachtungssituation. Darstellungen der Ergebnisse zu diesen Themenbereichen sind auf der Projektwebseite einsehbar: https://www.hs-kempten.de/bzpd-bayerisches-zentrum-pflege-digital/projekte/digitale-informations-und-unterstuetzungsangebote.

2 Pflegegradrechner sind meist in Webseiten integriert und sollen den Nutzenden eine Orientierung darüber geben, welcher Pflegegrad ihnen im Rahmen einer Begutachtung zugesprochen werden könnte.
